# Ciliated hepatic foregut cyst: A case report and review of literature

**DOI:** 10.1016/j.ijscr.2022.107356

**Published:** 2022-06-28

**Authors:** Cláudio Silva, Luísa Ferreira, Cláudio Branco, Vítor Simões, António Canha, Donzília Sousa Silva, Jorge Daniel, José Davide

**Affiliations:** a HEBIPA – Hepatobiliary and Pancreatic Unit, Department of General Surgery (Head: José Davide), Hospital de Santo António, Centro Hospitalar e Universitário do Porto EPE – Porto, Largo do Prof. Abel Salazar, 4099-001, Portugal; b Department of Pathology, Hospital de Santo António, Centro Hospitalar e Universitário do Porto EPE – Porto, Largo do Prof. Abel Salazar, 4099-001, Portugal

**Keywords:** Ciliated hepatic foregut cyst, Squamous cell carcinoma, Cyst enucleation, Liver

## Abstract

**Introduction:**

Ciliated hepatic foregut cyst (CHFC) is a rare cystic lesion that arises from the embryonic foregut with approximately 100 cases reported. Most commonly identified in segment IV of the liver, CHFC is typically asymptomatic and incidentally found on abdominal imaging. It is important to consider this entity in the differential diagnosis of atypical liver lesions since CHFC carries a risk of transformation into squamous cell carcinoma. A suspicion of CHFC is therefore an indication for surgical resection.

**Case presentation:**

A 62-year-old male presented to surgery consultation for further evaluation of a hepatic cyst incidentally found on abdominal ultrasound. The patient was completely asymptomatic. Both abdominal computerized tomography and magnetic resonance imaging scan confirmed a 4 cm subcapsular cyst in segment IVa. Additional workup was unremarkable. Considering the diagnostic doubt the patient underwent laparoscopic cyst enucleation. Histology revealed a ciliated pseudostratified epithelium consistent with a CHFC.

**Clinical discussion:**

CHFC is a rare diagnostic entity that should be considered in the differential diagnosis of cystic hepatic lesions, particularly those located in segment IV of the liver. Since it is frequently asymptomatic, CHFC is usually found incidentally during surgery or imaging studies. Diagnosis of CHFC preoperatively is difficult due to the lack of specific radiographic findings. Moreover, metaplasia and squamous carcinoma can occur. Therefore complete surgical excision is the recommended treatment.

**Conclusion:**

Despite its rarity, CHFC carries a risk of malignant transformation. Accurate diagnosis is mandatory and surgical excision is recommended even in asymptomatic CHFC.

## Highlights

• The ciliated hepatic foregut cyst is extremely rare, though increasingly being reported.
• CHFC is a benign cyst with potential for malignant transformation.
• Due to lack of evidence, management is not standardized.
• Complete surgical excision through a laparoscopic approach must be the treatment of choice.

## 1 Introduction

The ciliated hepatic foregut cyst (CHFC) is a benign cyst of the liver derived from an embryonic remnant of foregut epithelium [1]. Although it was first described in 1857 by Friedreich, only about 100 cases have been reported in the literature, with the largest single-center series describing 6 patients [2]. Even with the increasingly use of abdominal cross images and consequently the increasingly detection of incidental hepatic cysts, CHFC remains extremely rare [3]. Despite being uncommon, CHFC warrants special consideration due to its malignant potential [4], [5].

The CHFCs are frequently discovered incidentally during abdominal imaging. These cysts are often small, unilocular, subcapsular and located in segment IV of the left lobe of the liver. Typically, they do not communicate with the biliary system [4], [5]. These cysts have a characteristic four-layer border consisting of: an inner layer of ciliated, pseudostratified columnar epithelium; a subepithelial connective tissue; a smooth muscle band, ranging from one to three layers in thickness; and an outer fibrous capsule. The lining epithelium may secrete fluid of different composition (from watery to mucoid fluid) with subsequent variable echogenicity and intensity on imaging posing diagnostic challenges with other atypical hepatic lesions [1], [2], [3].

Although CHFCs are thought to follow a benign course, they yield a risk of transformation into squamous cell carcinoma (3–5 %) [4], [5]. Therefore, most authors defend that surgical resection should be indicated instead of long-term follow-up; even though management has not been standardized due to lack of evidence [1], [2], [3].

Herein, we report a clinical case of a CHFC discussing the challenges of its work-up and management. We also declare that this work is in line with the SCARE 2020 criteria [6].

## 2 Case presentation

**Fig. 1:**
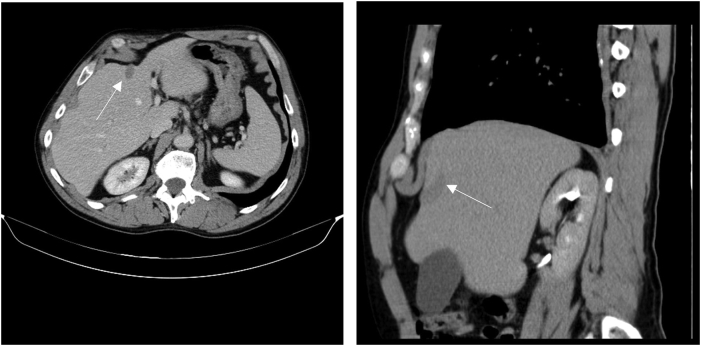
Transverse and sagittal abdominal computerized tomography of hepatic cyst located in segment IV of the liver (arrow) without contrast enhacement.

A 62-year-old male, with past history of smoking and pleural tuberculosis treated with antibiotics and several therapeutic thoracocentesis for symptomatic relief, was referred to surgery consultation in a tertiary hospital for further evaluation of a hepatic cyst. There was no family health history of interest. The patient was completely asymptomatic, and the cyst was incidentally found on routine abdominal ultrasound. On diagnostic work-up, aspartate transferase, alanine transaminase, gamma-glutamyl transferase, alkaline phosphatase, bilirubin and serological tumor markers (carcinoembryonic antigen, carbohydrate antigen 19-9, and alpha-fetoprotein) were unremarkable. Hepatotropic virus including hepatitis B, C and human immunodeficiency virus were excluded. The lesion was initially characterized with contrast abdominal computed tomography (CT) that revealed a 3.3 cm solitary lesion with low attenuation and without contrast enhancement located in the segment IV of the liver (Fig. 1).

**Fig. 2:**
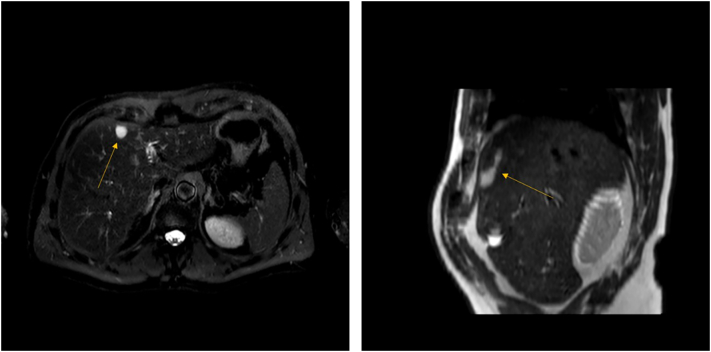
Transverse and sagittal abdominal magnetic resonance imaging of hepatic cyst located in segment IV of the liver (arrow).

A subsequent contrast enhanced abdominal magnetic resonance imaging (MRI) confirmed a 4.3 cm hypodense subcapsular cyst in segment IVa without enhancement or diffusion restriction raising the diagnostic suspicion of a CHFC (Fig. 2).

**Fig. 3:**
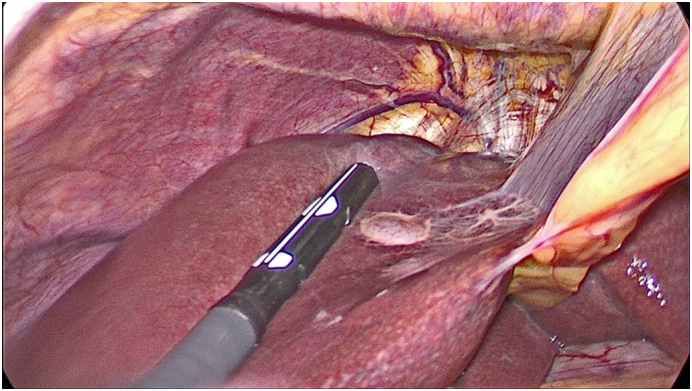
Intraoperative view of ciliated hepatic foregut cyst in segment IV.

Despite the benign appearance and considering the diagnostic doubt the decision was made to proceed with surgical resection. Written and signed consent was obtained from the patient prior to the procedure. The surgery was performed by a senior hepatobilliary surgeon through a laparoscopic approach, which revealed a superficial hepatic cyst within segment IV, consistent with prior imaging (Fig. 3).

A thin-walled with 3.5 × 2 × 1.5 cm cyst was removed via cyst enucleation without complications (video - https://vimeo.com/713880492). The post-operative recovery was uneventful, and the patient was discharged on post-operative day 2. At 3-month outpatient follow-up the patient was completely asymptomatic and satisfied with surgical outcomes.

**Fig. 4:**
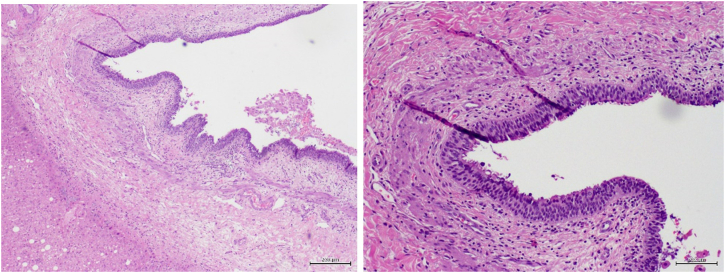
Histology of the cystic lesion with a pseudostratified ciliated epithelial lining, with loose connective tissue with a discontinuous smooth muscle layer of variable thickness and externally coated by fibrocollagenous tissue.

Histology of the surgical specimen revealed a well-circumscribed cyst measuring 3 × 2 × 1 cm with ciliated pseudostratified epithelium covered by subepithelial connective tissue, one smooth muscle layer and an outer fibrous capsule consistent with a CHFC (Fig. 4).

## 3 Discussion

Despite being increasingly reported, CHFC is still a rare entity. It is typically diagnosed during the fifth decade of life, but it can be found at any age. It is frequently asymptomatic and discovered incidentally during abdominal imaging. However, symptoms such as upper abdominal pain and jaundice have been reported, probably due to distention of the Glisson's capsule and compression of adjacent structures [1], [2], [3], [7].

The cyst is often located in the segment IV of the left lobe of the liver. However, other locations have been reported, namely right lobe of the liver and even extrahepatic locations such as the gallbladder. This lesion results from bronchial remain from the proximal intestine that becomes trapped usually in the liver during the early embryonic development [1], [8].

CHFC is usually hypoechoic on ultrasound, hypodense on CT and hyperintense on T2-weighted MRI; even though a variety of imaging findings has been described [9]. Therefore, there is a large differential diagnosis including simple cysts, pyogenic abscess, hydatid cyst, mucinous cystic neoplasm, biliary cystadenoma, and cystadenocarcinoma. As a result, combined imaging studies is recommended in order to increase diagnostic accuracy [1], [9].

Although diagnosis is suggested by findings in abdominal imaging, the definitive diagnosis is only made with histology by revealing a four-layer cyst with pseudostratified columnar epithelium [1], [9]. In addition, serological tumor markers (CEA, CA 19-9, AFP) seem to lack efficacy for diagnosis and showed no correlation with metaplasia or malignant transformation as they have been demonstrated to be elevated in benign CHFC and may be normal in malignancy [11]. Despite the ability of fine-needle aspiration biopsy to differentiate lesions bases on epithelial appearance or cyst contents, sampling error may lead to an errant diagnosis of benign CHFC in the face of missed malignant transformation.

Besides the diagnostic challenges that CHFC imposes, the management of CHFC remains controversial. Although it was initially thought to be a benign lesion, recent reports described CHFCs undergoing malignant transformation with squamous cell metaplasia, dysplasia and carcinoma being the most common transformation [1], [4], [5], [12]. To date, the major risk factor for the malignant transformation of a CHFC is its size with most of the cases occurring with cysts larger than 8 cm [4]. Other malignant predictive factors include worrying findings on imaging such as focal wall abnormalities or thick septations [4], [14] but malignant transformation can also occur in the absence of those findings. Therefore, most authors defend that surgical resection should be performed instead of long-term follow-up.

Laparoscopic excision is the preferred surgical approach due to the generally small size and the subcapsular and anterior location of CHFC that eases the removal of the lesion with minimal morbidity. Moreover, the commonly benign nature of CHFC allows excision without concern for tumor-free margin [13], [14]. After resection, long-term follow-up is not recommended since recurrence after treatment has never been described [1], [2].

## 4 Conclusion

CHFC is a rare diagnostic entity that should be considered in the differential diagnosis for cystic hepatic lesions, particularly those located in segment IV of the liver. Metaplasia and squamous carcinoma can occur, though rarely. Therefore, complete surgical excision through a laparoscopic approach is increasingly being recommended.

The following is the supplementary data related to this article.

Supplementary video Laparoscopic excision of ciliated hepatic foregut cyst. Supplementary video

## Provenance and peer review

Not commissioned, externally peer-reviewed.

## Sources of funding

None.

## Ethical approval

N/a.

## Consent

Written informed consent was obtained from the patient for publication of this case report and accompanying images. A copy of the written consent is available for review by the Editor-in-Chief of this journal on request.

## Research registration

N/a.

## Guarantor

Cláudio Silva (claudio.silva11@hotmail.com)

## CRediT authorship contribution statement

Cláudio Silva – data collection, data analysis and writing the paper.

Luísa Ferreira – assessed the histological specimen.

António Canha and Vítor Simões – performed the surgery and reviewed the draft.

Cláudio Branco, Donzília Sousa Silva, Jorge Daniel, José Davide – reviewed the draft

## Declaration of competing interest

None.

## References

[1] Ambe C., Gonzalez-Cuyar L., Farooqui S., Hanna N., Cunningham S.C. (2012). Ciliated hepatic foregut cyst: 103 cases in the world literature. Open J.Pathol..

[2] Bishop K.C., Perrino C.M., Ruzinova M.B., Brunt E.M. (2015). Ciliated hepatic foregut cyst: a report of 6 cases and a review of the English literature. Diagn. Pathol..

[3] Sharma S., Corn A., Kohli V., Wright H.I., Sebastian A., Jabbour N. (2008). Ciliated hepatic foregut cyst: an increasingly diagnosed condition. Dig. Dis. Sci..

[4] Wilson J.M., Groeschl R., George B., Turaga K.K., Patel P.J., Saeian K., Gamblin T.C. (2013). Ciliated hepatic cyst leading to squamous cell carcinoma of the liver–a case report and review of the literature. Int. J. Surg. Case Rep..

[5] Zhang X., Wang Z., Dong Y. (2009). Squamous cell carcinoma arising in a ciliated hepatic foregut cyst: case report and literature review. Pathol.Res.Pract..

[6] Agha R.A., Franchi T., Sohrabi C., Mathew G., for the SCARE Group (2020). The SCARE 2020 guideline: updating consensus Surgical CAse REport (SCARE) guidelines. Int. J. Surg..

[7] Kiyochi H., Okada K., Iwakawa K., Nkanishi M., Satoh H., Iimori S., Miyoshi M., Yamamoto K., Kotegawa H., Takai A., Kajiwara S. (2008). Ciliated hepatic foregut cyst with obstructive jaundice. Case Rep.Gastroenterol..

[8] Al Beteddini O.S., Amra N.K., Sherkawi E. (2016). Ciliated foregut cyst in the triangle of calot: the first report. Surg.Case Rep..

[9] Ansari-Gilani K., Modaresi Esfeh J. (2017). Ciliated hepatic foregut cyst: report of three cases and review of imaging features. Gastroenterol.Rep..

[11] Vick D.J., Goodman Z.D., Ishak K.G. (1999 Nov). Squamous cell carcinoma arising in a ciliated hepatic foregut cyst. Arch. Pathol. Lab. Med..

[12] de Lajarte-Thirouard A.S., Rioux-Leclercq N., Boudjema K., Gandon Y., Ramée M.P., Turlin B. (2002). Squamous cell carcinoma arising in a hepatic forgut cyst. Pathol.Res.Pract..

[13] Goodman M.D., Mak G.Z., Reynolds J.P., Tevar A.D., Pritts T.A. (2009). Laparoscopic excision of a ciliated hepatic foregut cyst. J.Soc.Laparoendosc.Surg..

[14] Saravanan J., Manoharan G., Jeswanth S., Ravichandran P. (2014). Laparoscopic excision of large ciliated hepatic foregut cyst. J.Minim.Access Surg..

